# Digital screen exposure and emotional symptoms in preschool children: mediation by parent–child relationship and moderation by peer relationships

**DOI:** 10.3389/fpsyg.2025.1584919

**Published:** 2025-05-09

**Authors:** Yongqi Xu, Lei Qiao

**Affiliations:** ^1^School of Education, Minzu University of China, Beijing, China; ^2^Academic Affairs Office, Pu’er University, Pu’er, Yunnan, China

**Keywords:** digital screen exposure, emotional symptoms, parent–child relationship, peer relationships, preschool children

## Abstract

**Introduction:**

With the rapid development of information technology, emerging electronic media are widely used in various settings where children are present. At the same time, excessive screen exposure has been associated with various emotional symptoms in preschool children.

**Methods:**

This study employed a questionnaire survey to collect data from 7,239 parents of preschool children in Pu’er City, China. After rigorous data screening, 6,623 valid responses were retained for analysis. The collected data were then analyzed using SPSS 25.0 for descriptive and inferential statistics, and Hayes’ PROCESS 4.0 was used to test the mediation and moderation models involving digital screen exposure, the parent-child relationship, peer relationships, and emotional symptoms in preschool children.

**Results:**

The results indicated that digital screen exposure was significantly associated with emotional symptoms (β = 0.2351, *p* < 0.01). Specifically, higher levels of screen exposure were associated with more severe emotional symptoms, and this association was mediated by the parent-child relationship (indirect effect = 0.06, 95% CI [0.03, 0.08]). Peer relationships moderated the association between digital screen exposure and emotional symptoms (interaction effect = −0.22, *p* < 0.01) and between digital screen exposure and the parent-child relationship (interaction effect = −0.12, *p* < 0.01).

**Discussion:**

This study provides a comprehensive theoretical framework to understand the associations among digital screen exposure, emotional symptoms, and social relationships in preschool children. It highlights the potential importance of the parent-child relationship and peer relationships in buffering negative associations related to digiatl screen exposure.

## Introduction

Digital screen exposure refers to individuals’ engagement with various screen-based electronic media (Slater, 2004). In recent years, the rapid development of information technology has led to the deep integration of electronic media into family life. Emerging media such as smartphones and tablets, along with traditional media like televisions and computers, have collectively shaped the media ecosystem in which children grow up. Studies indicate that 98% of preschool children have been exposed to at least one type of electronic media before the age of four, with most beginning exposure as early as 1 year old (Kabali et al., 2015). In addition to the early onset of exposure, the duration of daily electronic media use is also concerning, as most preschool children spend more than 2 h per day on screens (You et al., 2023). This phenomenon has garnered widespread attention in academia. Children’s emotional health is a crucial component of their overall development. Strong emotional regulation skills enable children to better express their feelings and understand the emotions of others (Morrison and Grammer, 2016). However, increasing research suggests that excessive screen exposure may be negatively associated with children’s emotional development, potentially contributing to issues such as heightened anxiety, emotional instability, and social withdrawal (Bustamante et al., 2023; Wu et al., 2023). Furthermore, a lack of effective media usage guidance may be associated with unhealthy media habits, potentially exacerbating these negative associations (Guellai et al., 2022). Although previous studies have explored the associations between digital screen exposure and children’s emotional development, the underlying mechanisms remain unclear. According to Family Systems Theory (Bowen, 1994), the family is viewed as an emotional system, where changes in one member’s behavior (such as increased screen exposure) may be associated with changes across the whole system, potentially influencing interactions and emotional health among family members. Do different types of screen content show varying associations with children’s emotions? Does the parent–child relationship play a crucial role in this association? Can peer relationships buffer potential negative associations with screen exposure? To address these questions, this study systematically examines the associations among digital screen exposure, the parent–child relationship, peer relationships, and emotional symptoms, aiming to provide scientific evidence for parents and educators to promote children’s healthy development.

### Digital screen exposure and children’s emotional symptoms

Emotional symptoms in preschool children refer to abnormal emotional and behavioral responses observed in children aged 0 to 6 years (Klein et al., 2015). These symptoms may include anxiety, fear, depression, mood swings, lack of motivation, and social withdrawal (Carneiro et al., 2019). Existing studies suggest a significant association between digital screen exposure and emotional symptoms in preschool children, with higher levels of digital screen exposure associated with increased levels of anxiety, depression, and emotional instability (Skalická et al., 2019; Liu et al., 2024). Research also indicates that excessive digital screen exposure is associated with attention difficulties, fatigue, and impaired self-regulation in young children (Cliff et al., 2018; Cerniglia et al., 2021). Moreover, the type of screen content plays an important role. For example, exposure to violent, fast-paced, or highly stimulating content has been associated with higher levels of anxiety and aggressive behaviors (Liu et al., 2024), while educational or prosocial content has been related to neutral or positive associations (Coyne et al., 2022). Additionally, studies have shown that screen exposure, especially before bedtime, may be associated with disrupted sleep quality, which in turn is associated with emotional symptoms such as irritability and mood instability (Brockmann et al., 2016; Montagni et al., 2016).

Based on these findings, this study proposes the following hypothesis:

*H1*: Digital screen exposure is positively associated with emotional symptoms in preschool children.

### The mediating role of the parent–child relationship

The parent–child relationship refers to the emotional bond, communication patterns, and the quality of interactions between parents and their children (Pianta, 1994). According to Family Systems Theory, the parent–child relationship is a critical subsystem within the family, where emotional dynamics may be associated with both individual well-being and family functioning. Family Systems Theory further suggests that internal family interactions, particularly the parent–child relationship, could serve as important mediating mechanisms linking family members’ behaviors (such as digital screen exposure) with children’s emotional and behavioral outcomes (Bowen, 1994). Existing research indicates that digital screen exposure may be negatively associated with the parent–child relationship. Specifically, excessive screen time may be associated with fewer opportunities for meaningful interactions, such as shared play, conversations, and emotional support, potentially weakening emotional bonds and communication quality between parents and children (Guerrero et al., 2019; Lu and Li, 2022). Moreover, increased screen exposure may also be related to reduced parental responsiveness and emotional availability, as parents might be less engaged in their children’s emotional and behavioral needs when digital devices dominate family routines (Coyne et al., 2023; Liu et al., 2021). These potential disruptions in the parent–child relationship, in turn, may be associated with difficulties in children’s emotional regulation, potentially contributing to emotional symptoms such as anxiety, depression, and irritability. Therefore, the parent–child relationship may serve as a key mediating mechanism through which digital screen exposure is associated with children’s emotional well-being.

Building on these findings, this study proposes the following hypothesis:

*H2*: The parent-child relationship mediate the association between digital screen exposure and preschool children's emotional symptoms.

### The moderating role of peer relationships

Peer relationships refer to the social connections and interactions that children establish with their peers, including friendships, group affiliations, and social acceptance within peer groups (Durkin, 2010). Family Systems Theory suggests that external systems (such as peer relationships) may interact with family subsystems (such as the parent–child relationship) to jointly relate to children’s developmental outcomes (Bowen, 1994). According to this theory, peer relationships can function as external protective factors that moderate or buffer the associations between family-related stressors (e.g., increased digital screen exposure, weakened parent–child relationship) and children’s emotional difficulties, particularly when internal family support is limited or impaired. First, peer relationships may help buffer potential negative associations between digital screen exposure and emotional symptoms. Studies have shown that positive peer relationships may provide emotional support and social interaction, potentially helping children manage negative emotions associated with screen exposure, such as anxiety and loneliness, through effective emotional expression and regulation strategies (Livingstone et al., 2018). Second, peer relationships may moderate the association between digital screen exposure and the parent–child relationship. When the parent–child relationship is weaker in contexts of high digital screen exposure, strong peer relationships may compensate for deficiencies in the parent–child bond, potentially alleviating tension and conflict, and thereby buffering negative associations related to digital screen exposure (Liu et al., 2021). Third, peer relationships may also moderate the association between the parent–child relationship and emotional symptoms. Research suggests that when the parent–child relationship is weak, positive peer relationships may partially buffer negative associations with children’s emotional symptoms by facilitating social interaction and emotional support, thus potentially enhancing children’s emotion regulation abilities (Lindsey, 2017). Moreover, it is important to consider the potential interaction between the parent–child relationship and peer relationships in relation to children’s emotional outcomes. For instance, a strong parent–child relationship may buffer potential negative associations related to negative peer experiences, while a supportive peer environment might compensate for potential deficiencies in the parent–child relationship (Curby et al., 2022).

Building on these findings, this study proposes the following hypothesis:

*H3*: Peer relationships moderate the association between digital screen exposure, parent-child relationships, and preschool children's emotional symptoms.

### The present study

**Figure 1:**
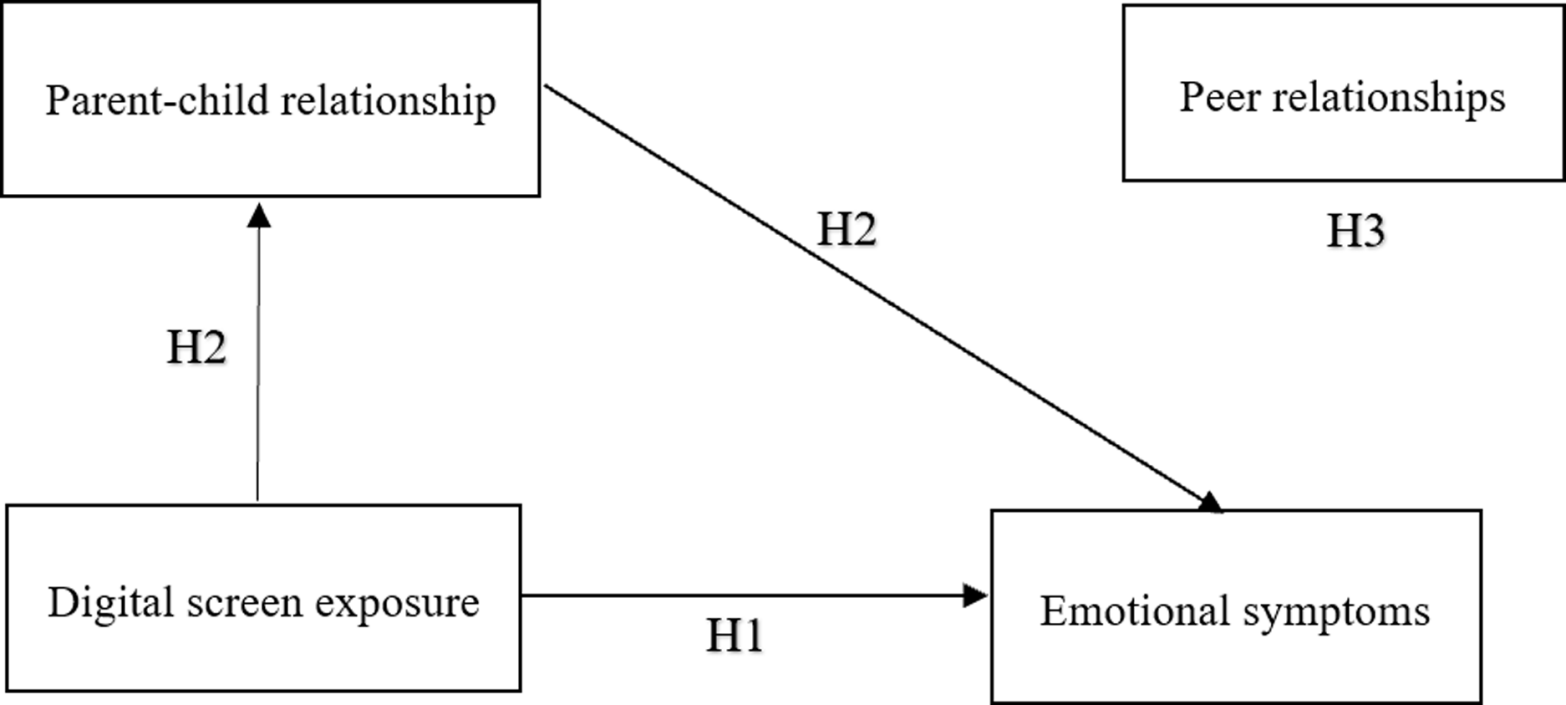
The hypothetical modle.

While existing research has examined the associations between electronic media, particularly digital screen exposure, and children’s emotional symptoms, the underlying mechanisms through which these associations exist remain unclear. Most studies have focused on various influencing factors, such as parental influences and child-related variables, yet there is still a lack of comprehensive investigation into how digital screen exposure is specifically associated with the emotional development of preschool children. Incorporating Family Systems Theory into this research, the study recognizes that the interplay between internal family dynamics (the parent–child relationship) and external social systems (peer relationships) may provide a more comprehensive understanding of how digital screen exposure relates to emotional development. Therefore, the primary objective of this study is to explore the associations among digital screen exposure, emotional symptoms, the parent–child relationship, and peer relationships in preschool children. To this end, the study posits the following hypotheses: (H1) Digital screen exposure is positively associated with preschool children’s emotional symptoms; (H2) The parent–child relationship mediates the association between digital screen exposure and preschool children’s emotional symptoms; (H3) Peer relationships moderate the association between digital screen exposure, parent–child relationships, and preschool children’s emotional symptoms (see Figure 1).

## Methods

### Participants

This study conducted a questionnaire survey among 7,239 parents of preschool children in Pu′er City, China. The survey was conducted through anonymous questionnaire completion, and strict screening criteria were applied to ensure data quality. The screening process included the following criteria: (1) Incomplete responses, where questionnaires with missing key information were excluded; (2) Unrealistic response times, meaning questionnaires completed in an excessively short time, suggesting a lack of careful reading, were removed; (3) Identical or patterned responses, such as selecting the same option for all questions, indicating low response reliability; (4) Logical inconsistencies, where contradictory answers were detected in different sections of the questionnaire; and (5) Duplicate submissions, where multiple identical responses from the same participant were identified. After applying these criteria, 6,623 valid questionnaires were obtained. The final sample comprised 3,462 boys (52.3%) and 3,161 girls (47.7%) aged 3–6 years (*M* = 4.7 years), with balanced representation across urban (*n* = 4,106) and rural (*n* = 2,517) households. Participating parents included 1,880 fathers (28.4%), 4,569 mothers (69.0%), and 174 other guardians (2.6%). Household monthly income was categorized as: <3,000 CNY (15.2%), 3,000–5,000 CNY (32.8%), 5,001–10,000 CNY (29.7%), and >10,000 CNY (22.3%) based on 2023 regional income percentiles. All procedures were approved by the Institutional Review Board (IRB) of the first author’s institution. All measurements and procedures were permitted by the Institutional Review Board (IRB) of the first author’s institution.

### Measures

All the questionnaires used in this study were adapted from well-established scales. To ensure linguistic accuracy and cultural appropriateness while maintaining the original structure and conceptual framework, we made only minor wording adjustments during the translation process. First, two doctoral researchers in educational psychology translated the questionnaires. Next, two experts in the same field reviewed the translations to ensure conceptual consistency with the original scales. Additionally, while these scales have been widely validated in previous studies, we acknowledge the importance of reassessing their validity in our specific sample. Therefore, we have conducted confirmatory factor analysis (CFA) and reported construct validity indices [including Average Variance Extracted (AVE) and Composite Reliability (CR)]. These results confirm that the adapted scales retain strong validity in the current study.

### The digital screen exposure

The study utilized the digital screen exposure questionnaire developed by Kaur et al. (2021). The questionnaire consists of 27 items and includes three dimensions: screen exposure time, media-related behaviors, and family media environment. It includes questions such as “Frequency of performing the activity in a given week (Watches TV, Uses a smartphone, Uses other gadgets),” “The child uses video calling applications to talk to family/friends,” and “The child plays video games.” The questionnaire uses a 1–5 scoring scale, ranging from 1 (strongly disagree) to 5 (strongly agree). The average score is calculated as the final score, with higher scores indicating more severe digital screen exposure for the child. The Cronbach’s alpha coefficient for the sub-dimensions of this questionnaire demonstrated good internal consistency: 0.742 for screen exposure time, 0.725 for media-related behaviors, and 0.710 for family media environment. Regarding convergent validity, the Average Variance Extracted (AVE) for Digital screen exposure was 0.51, which is slightly above the threshold of 0.5, indicating that the construct explains more than half of the variance in its indicators. The Composite Reliability (CR) was 0.81, which is above the acceptable threshold of 0.7, suggesting good internal consistency and reliability.

### The parent–child relationship

The study utilized Pianta’s Parent–Child Relationship Scale, which includes three dimensions: closeness, conflict, and dependency (Pianta, 1994). The scale contains 26 items, including questions such as “My child easily becomes angry at me,” “If upset, my child will seek comfort from me,” and “My child is overly dependent on me.” The questionnaire uses a 1–5 scoring scale, ranging from 1 (strongly disagree) to 5 (strongly agree). A higher score in the closeness dimension indicates a better parent–child relationship, while higher scores in the dependency and conflict dimensions indicate a poorer relationship. To facilitate statistical analysis, the dependency and conflict dimensions were reverse scored and then summed with the closeness scores. The average score was calculated as the final score, with higher scores indicating a worse parent–child relationship. The Cronbach’s alpha coefficients for the closeness, conflict, and dependency dimensions were 0.767, 0.725, and 0.714, respectively. For convergent validity, the Average Variance Extracted (AVE) for the Parent–child Relationship construct was 0.62, and the Composite Reliability (CR) was 0.87, both indicating good reliability and convergent validity of the scale in measuring parent–child relationships.

### The peer relationships

The assessment of peer relationships was based on Goodman’s Strengths and Difficulties Questionnaire, which includes five items related to peer relationships (Goodman, 1997). These items include questions such as “Has at least one good friend,” “Generally liked by other children,” and “Gets on better with adults than with other children.” The questionnaire uses a 1–5 scoring scale, ranging from 1 (strongly disagree) to 5 (strongly agree). The average score was calculated as the final score, with higher scores indicating better peer relationships. The Cronbach’s alpha coefficient for this questionnaire was 0.730. For convergent validity, the Average Variance Extracted (AVE) for the Peer Relationship construct was 0.52, and the Composite Reliability (CR) was 0.79, indicating good reliability and convergent validity of the scale in assessing peer relationships.

### The emotional symptoms

The assessment of emotional symptoms was based on Goodman’s Strengths and Difficulties Questionnaire, which includes five items related to emotional symptoms (Goodman, 1997), such as “Many fears, easily scared,” “Many worries, often seems worried,” and “Nervous or clingy in new situations, easily loses confidence.” The questionnaire uses a 1–5 scoring scale, ranging from 1 (strongly disagree) to 5 (strongly agree). The average score was calculated as the final score, with higher scores indicating more severe emotional symptoms. The Cronbach’s alpha coefficient for this questionnaire was 0.703. For convergent validity, the Average Variance Extracted (AVE) for the Emotional Symptoms construct was 0.57, and the Composite Reliability (CR) was 0.83, indicating good reliability and convergent validity of the scale in assessing emotional symptoms.

## Results

This study employed SPSS 25.0 and Hayes’ PROCESS 4.0 to examine a moderated mediation model. Initially, common method bias was assessed and descriptive statistics were conducted using SPSS. Additionally, correlations among digital screen exposure, parent–child relationship, peer relationship, and emotional symptoms were analyzed. Subsequently, PROCESS Model 4 was utilized to examine the mediating effect of parent–child relationship, while PROCESS Model 59 was employed to investigate the moderating effect of peer relationship.

### Common method bias

Due to the fact that the core variables in this study were all measured through parental questionnaires, there may be a potential common method bias. Therefore, a common method bias test is necessary before data analysis. Firstly, in order to mitigate the influence of common method bias, anonymous response and reverse scoring methods were employed during questionnaire design. Secondly, through the Harman single-factor test, it was found that there were six factors with eigenvalues greater than 1, and the variance explained by the largest factor was 21.6% (less than 40%). Hence, the common method bias in this study is not expected to significantly affect the relationships among variables.

### Descriptive and correlation statistics

Through correlation analysis (refer to Table 1), it was found that there were significant positive correlations among children’s digital screen exposure, the parent–child relationship, and emotional symptoms. Additionally, a significant positive correlation was observed between peer relationships and digital screen exposure, while a significant negative correlation was found between peer relationships and the parent–child relationship, as well as between peer relationships and emotional symptoms. The study also indicated a significant negative correlation between emotional symptoms and age. Furthermore, significant positive correlations were found between digital screen exposure, peer relationships, and gender, whereas a significant negative correlation was observed between the parent–child relationship and gender. Additionally, the study conducted tests for multicollinearity, revealing that the VIF values for all variables were below 10, indicating minimal to no multicollinearity among the variables.

**Table 1:** Means, standard deviations, and correlations of the variables (*N* = 6,623).

Variables	*M*	SD	1	2	3	4	5	6
Age	2.69	0.94	1					
Gender	1.47	0.50	−0.03^*^	1				
Digital screen exposure	3.35	0.69	−0.08	0.04^**^	1			
Parent–child relationship	2.61	0.40	0.02	−0.03^*^	0.10^**^	1		
Peer relationship	2.4	0.70	0.21	0.07^**^	0.35^***^	−0.37^**^	1	
Emotional symptoms	3.93	0.53	−0.03^*^	0.01	0.23^**^	0.58^**^	−0.30^**^	1

^*^p < 0.05, ^**^p < 0.01, ^***^p < 0.001.

### The mediating effect of parent–child relationship

This study examined the mediating effect of the parent–child relationship using Model 4 in the SPSS PROCESS macro (refer to Tables 2, 3). The results revealed that digital screen exposure was significantly positively associated with emotional symptoms (*β* = 0.24, *t* = 19.35, *p* < 0.001). When introducing the mediator variable (parent–child relationship), the relationship between digital screen exposure and emotional symptoms remained significant (β = 0.18, *t* = 17.80, *p* < 0.01). Additionally, digital screen exposure was significantly positively associated with parent–child relationship (β = 0.06, *t* = 8.21, *p* < 0.01), while the parent–child relationship was significantly positively associated with emotional symptoms (*β* = 0.10, t = 57.72, *p* < 0.01). According to Table 3, it is evident that the bootstrap 95%CI for the direct effect of digital screen exposure on emotional symptoms does not include 0 [effect = 0.18, 95% CI (0.15, 0.21)], as well as the bootstrap 95% CI for the mediating effect of parent–child relationship [effect = 0.06, 95% CI (0.03, 0.08)]. This indicates that digital screen exposure was significantly associated with children’s emotional symptoms through the mediating role of the parent–child relationship. Specifically, the direct effect and the mediating effect account for 75.40 and 24.60% of the total effect, respectively.

**Table 2:** The mediation model analysis.

Regression equation (*N* = 999)		Fitting index			Coefficient significance	
Predictor variable	Outcome variable	*R*	*R* ^2^	*F*	β	*t*
Digital screen exposure	Emotional symptoms	0.23	0.05	374.24	0.24	19.35^***^
Digital screen exposure	Parent–child relationship	0.10	0.01	67.43	0.06	8.21^**^
Parent–child relationship Digital screen exposure	Emotional symptoms	0.61	0.37	1947.24	0.10 0.18	57.72^**^ 17.80^**^

^**^p < 0.01, ^***^p < 0.001, Bootstrap SEs and 95% CIs for mediating effects are reported in Table 3.

**Table 3:** Analysis of total effect, direct effect, and mediating effect.

	Effect	Boot SE	Boot LLCI	Boot ULCI	Percentage of in effect value
Total effect	0.24	0.01	0.21	0.26	
Direct effect	0.18	0.01	0.15	0.21	75.40%
Mediating effect of parent–child relationship	0.06	0.01	0.03	0.08	24.60%

### The moderating effect of peer relationships

**Figure 2:**
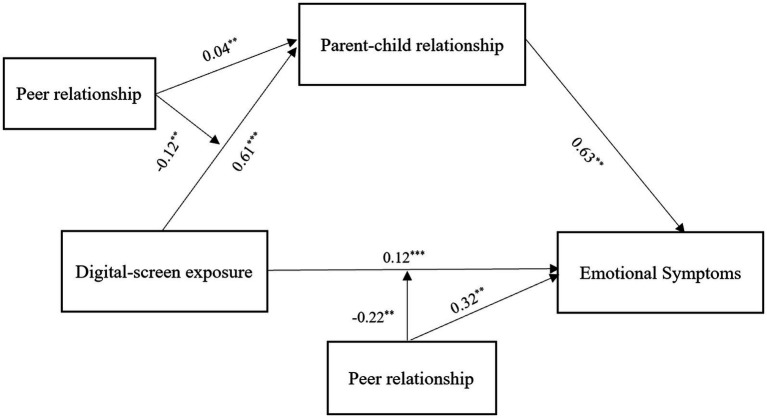
The moderated mediation model.

This study examined the moderating effect of peer relationships using Model 59 in Hayes’ process macro (refer to Table 4). The results revealed that peer relationships significantly moderated the association between digital screen exposure and the parent–child relationship (*β* = −0.12, *t* = −16.12, *p* < 0.01). Additionally, peer relationships moderated the association between digital screen exposure and emotional symptoms (β = −0.22, *t* = −17.50, *p* < 0.01). However, the moderating effect of peer relationships on the association between parent–child relationship and emotional symptoms was not significant (β = 0.04, *t* = 1.70, *p* > 0.05). Thus, the study validated two pathways as hypothesized in the theoretical framework. The modified moderated mediation model with mediation is presented in Figure 2.

**Table 4:** The moderated mediation model analysis.

Regression equation (*N* = 999)		Fitting index			Coefficient significance	
Outcome variable	Predictor variable	*R*	*R^2^*	*F*	*β*	*T*
Parent–child relationship	Digital screen exposure	0.48	0.23	659.65^**^	0.61	20.94^***^
	Peer relationship				0.04	1.58
	Digital screen exposure × Peer relationship				−0.12	−16.12^**^
Emotional symptoms	Digital screen exposure	0.66	0.43	1002.32^**^	0.12	22.18^***^
	Parent–child relationship				0.63	6.78^**^
	Peer relationship				0.32	5.59^**^
	Digital screen exposure × Peer relationship				−0.22	−17.50^**^
	Parent–child relationship × Peer relationship				0.04	1.70

^**^p < 0.01, ^***^p < 0.001, Bootstrap SEs and 95% CIs for moderation effects are reported in Table 5.

**Figure 3:**
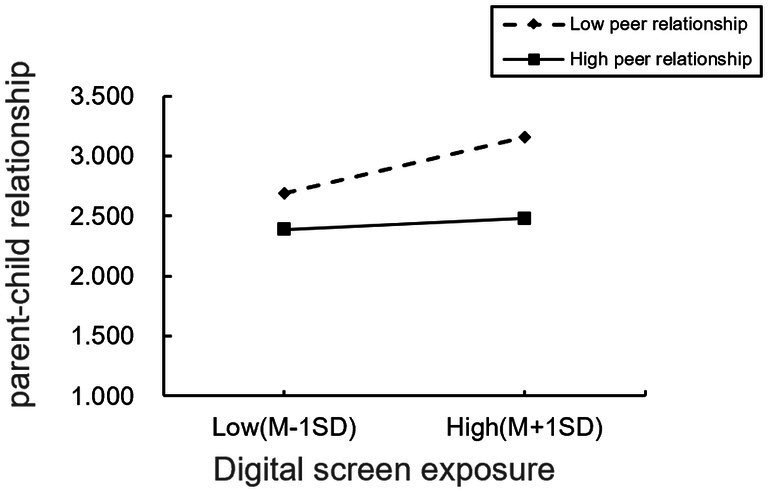
The moderating effect of peer relationship 1.

**Figure 4:**
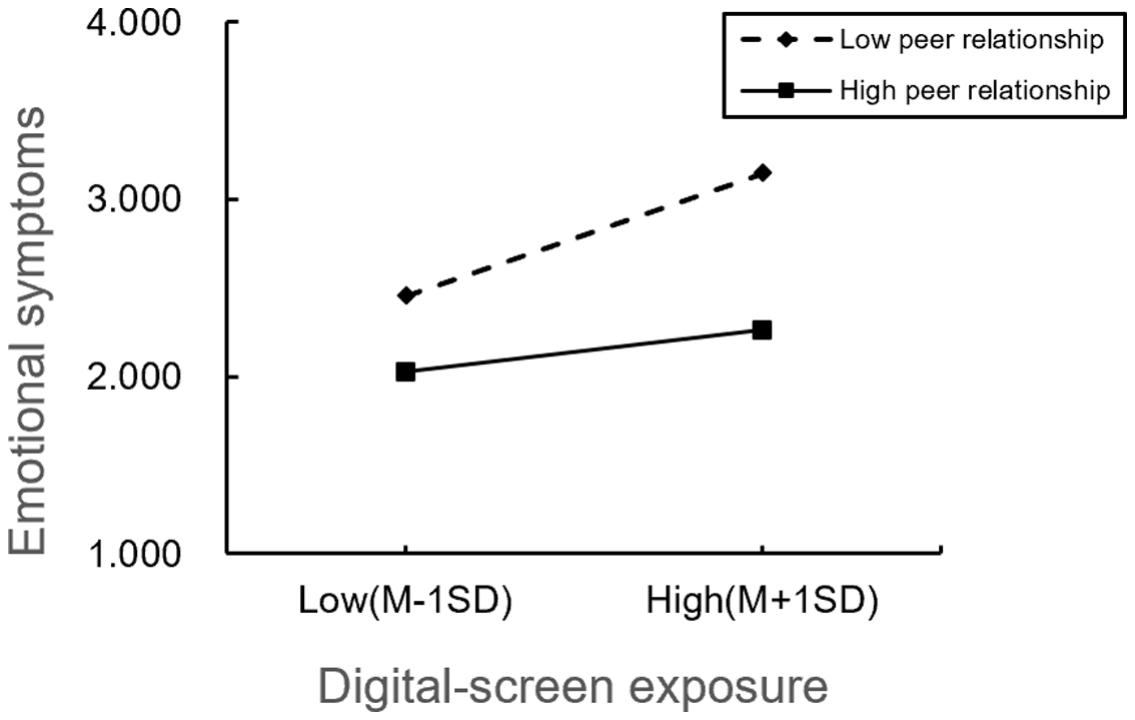
The moderating effect of peer relationship 2.

To further elucidate the moderating effect of peer relationships, the study conducted simple slope tests (refer to Figures 3, 4). As depicted in Figure 3, it is evident that for participants with lower levels of peer relationships, digital screen exposure was significantly positively associated with parent–child relationship (simple slope = 0.20, *t* = 27.29, *p* < 0.01). However, for participants with higher levels of peer relationships, although digital screen exposure also remained positively associated with the parent–child relationship, the strength of this association was reduced (simple slope = 0.07, *t* = 7.74, *p* < 0.01). This indicates that as the level of peer relationships strengthen, the association between digital screen exposure and parent–child relationship becomes weaker.

According to Figure 4, it is evident that for participants with lower levels of peer relationship, digital screen exposure was significantly positively associated with children’s emotional symptoms (simple slope = 0.50, *t* = 40.94, *p* < 0.01). However, for participants with higher levels of peer relationships, the positive association between digital screen exposure and children’s emotional symptoms was less pronounced (simple slope = 0.17, *t* = 11.68, *p* < 0.01). This indicates that as the level of peer relationships strengthen, the association between digital screen exposure and children’s emotional symptoms diminishes. Additionally, across three different levels of peer relationships, the mediating effect of the parent–child relationship also exhibits a decreasing trend (refer to Table 5). This suggests that with the strengthening of peer relationships, digital screen exposure is less likely to induce children’s emotional symptoms through its impact on parent–child relationship.

**Table 5:** Direct and mediated effects on different levels of peer relationships.

	Peer relationship	Effect	Boot SE	Boot LLCI	Boot ULCI
Direct effect	−1(M−1SD)	0.35	0.01	0.32	0.38
	0(M)	0.23	0.01	0.21	0.26
	1(M+1SD)	0.11	0.01	0.08	0.15
The mediating role of parent–child relationship	−1(M−1SD)	0.15	0.01	0.13	0.18
	0(M)	0.10	0.01	0.08	0.12
	1(M+1SD)	0.05	0.01	0.03	0.08

## Discussion

### The association between digital screen exposure and preschool children’s emotional symptoms

Research findings indicate that digital screen exposure is significantly positively associated with emotional symptoms in preschool children, meaning that higher screen exposure levels are associated with more severe emotional problems. This finding aligns with existing studies (Eirich et al., 2022; Hinkley et al., 2014; Wu et al., 2023).

To begin with, the duration of screen exposure may affect children’s emotional regulation abilities. Prolonged use of electronic devices has been associated with to reduced attention span and increased fatigue, potentially weakening children’s ability to regulate emotions and making them more prone to anxiety and irritability (Cerniglia et al., 2021). Since the preschool years are a critical period for emotional development, excessive screen exposure may impair self-regulation, making it difficult for children to manage negative emotions effectively (Przybylski and Weinstein, 2019).

Moreover, the nature of screen content is another crucial factor potentially influencing children’s emotions. Violent, fast-paced, highly stimulating, or socially negative content may trigger anxiety, fear, and even aggressive behavior in children (Liu et al., 2024). Given preschoolers’ underdeveloped cognitive abilities, they may struggle to differentiate between virtual and real-life scenarios, making them more susceptible to distress or imitation of aggressive behaviors (Keşşafoğlu et al., 2024). Even educational content, if consumed excessively, may be related to reduced opportunities for children to develop real-life emotional coping skills (Coyne et al., 2022).

Additionally, the association between screen exposure and sleep problems is a significant contributor to children’s emotional symptoms. Digital screen exposure, particularly before bedtime, has been linked to disrupted circadian rhythms and decreased sleep quality, which in turn have been associated with emotional instability and heightened anxiety (Aishworiya et al., 2018; Kahn et al., 2021). Sleep deprivation not only appears to impair emotional regulation but also negatively affects children’s overall physical and mental development.

### The mediating effect of parent–child relationship

This study found that parent–child relationships mediate the association between digital screen exposure and emotional symptoms in preschool children. That is, digital screen exposure is associated with the quality of parent–child relationship, which in turn is related to children’s emotional well-being. This conclusion is consistent with previous studies (Guerrero et al., 2019; Coyne et al., 2023; Laugen et al., 2024).

First, digital screen exposure may be associated with a weaker parent–child relationship, thereby indirectly relating to children’s emotions. When children spend extended periods engaged in screen activities, the time and quality of parent–child interactions may decline, which is associated with weaker emotional bonds (Laugen et al., 2024). Research has shown that high levels of digital screen exposure in families are often associated with reduced parent–child communication, lower emotional support, and increased parenting stress (Skalická et al., 2019), all of which have been associated with heightened emotional symptoms such as anxiety and depression in children.

Second, the quality of the parent–child relationship plays a crucial mediating role in the association with children’s emotional regulation. In cases of high screen exposure, if the parent–child relationship is poor, children may experience insufficient emotional support and parental supervision, making them more vulnerable to potential negative consequences associated with screen content, such as social withdrawal, impulsive behavior, or emotional dysregulation (Huang et al., 2024). Conversely, when the parent–child relationship is strong, parents may be better able to actively guide their children’s digital screen exposure through regulation, selecting appropriate content, and compensating for potential social deficits associated with digital media through quality interactions. This, in turn, may help protect children’s emotional regulation abilities (Liu et al., 2021).

It should be noted that the direct association of digital screen exposure with children’s emotional symptoms accounted for approximately 75.4% of the total association, indicating a substantial direct link beyond the mediation of the parent–child relationship (24.6%). This phenomenon may occur because emotional symptoms in preschool children could also be directly associated with factors inherently linked to digital screen exposure, such as overstimulation, disrupted sleep patterns, or exposure to inappropriate content, independent of parental interaction quality (Cerniglia et al., 2021; Liu et al., 2024). Therefore, while the parent–child relationship significantly mediates this association, the dominant direct association highlights potential inherent risks associated with excessive screen exposure itself. Future research should further explore other potential mediating mechanisms, such as cognitive development and physiological factors, to provide a more comprehensive understanding of these direct associations.

### The moderating effect of peer relationships

Research findings indicate that peer relationships moderate the association between digital screen exposure and emotional symptoms in preschool children, potentially buffering its negative associations. This aligns with previous studies (Livingstone et al., 2018; Ortega-Mohedano and Pinto-Hernández, 2021; Curby et al., 2022).

As shown in Figure 2, peer relationships exert a moderating effect on two pathways. First, they moderate the association between digital screen exposure and the parent–child relationship. Strong peer relationships may provide emotional support and social engagement, helping children regulate emotions and potentially mitigate behavioral issues even in contexts of high screen exposure (Ma and Zhang, 2020). Through peer interactions, children may develop better conflict resolution and emotional expression skills, which could compensate for a weakened parent–child relationship (Liu et al., 2021). Conversely, negative peer experiences, such as rejection, may be associated with increased digital screen exposure as an emotional escape, potentially intensifying negative emotional symptoms associated with digital exposure (Wu et al., 2017). In such cases, the protective role of the parent–child relationship may be insufficient.

Second, peer relationships directly moderate the association between screen exposure and children’s emotional symptoms. Positive peer connections may be associated with enhanced resilience, helping children cope with stress potentially related to screen exposure. Engaging with peers in shared screen activities may foster social interaction, which could help reduce loneliness and anxiety (Shoshani et al., 2022). In contrast, children lacking peer support may be more likely to engage in screen activities as a substitute for social connection, potentially reinforcing a cycle of excessive digital screen exposure and emotional distress (Evans et al., 2022).

However, peer relationships did not moderate the association between the parent–child relationship and emotional symptoms. This may be because parent–child interactions occur in a different relational context from peer dynamics. The parent–child relationship may shape fundamental emotional security and attachment, which are relatively stable and less likely to be influenced by external peer interactions. While peers contribute to social development, they likely do not replace the foundational role of parents in children’s emotional well-being. Thus, peer relationships may buffer associations between screen exposure and emotional symptoms but might not significantly alter the association between the parent–child relationship and emotional outcomes.

By comparing the findings of this study with previous research, we found evidence consistent with existing studies. However, a notable new finding of our research is the clear identification of peer relationships as a moderator of the association between digital screen exposure and children’s emotional symptoms. Although prior literature has recognized the potential benefits associated with peer support (Livingstone et al., 2018; Liu et al., 2021), our study quantitatively demonstrates the buffering role peer relationships may play in the association between digital screen exposure and emotional symptoms, thus offering new directions for future intervention strategies. Additionally, this study identifies the moderating role of peer relationships in the association between digital screen exposure and the parent–child relationship, further enriching our understanding of the complex interplay among individual, familial, and social factors that may be associated with children’s emotional development.

## Limitations and future directions

This study has some limitations. First, the core variables included are limited. This research focuses on the parent–child relationship as a mediating variable and peer relationships as a moderating variable to explore the association between digital screen exposure and preschool children’s emotional symptoms. However, considering the multifaceted nature of factors influencing emotional symptoms in preschool children, future research should integrate a broader range of variables for a comprehensive analysis. Second, there are limitations related to the use of parental questionnaires. Given the age of the preschool children, this study primarily collected data through parental questionnaires, which may lead to sample bias. Third, the cross-sectional design limits causal interpretations. This study is cross-sectional and does not examine preschool children’s emotional symptoms over time. Therefore, future research should continue with longitudinal studies to further investigate the directionality of these associations. Fourth, our sample was geographically restricted to Pu’er City, China, which may limit generalizability to populations with distinct cultural or socioeconomic characteristics (e.g., urban vs. rural disparities, varying access to digital devices). Future replication studies should employ stratified sampling across diverse regions.

## Conclusion

This study explored the mechanisms associated with the relationship between digital screen exposure and emotional symptoms in preschool children and tested the related hypothesized model. The results indicate that digital screen exposure is significantly positively associated with emotional symptoms in preschool children, with the parent–child relationship identified as a mediating factor and peer relationships identified as a moderating factor. This study provides a more comprehensive theoretical framework that highlights the potential importance of social factors associated with emotional regulation. This framework offers new perspectives for future research, encouraging scholars to further investigate how different social relationships may be related to children’s emotional adaptation in digital environments. To support children’s emotional well-being in the digital era, multiple stakeholders should consider proactive measures. Parents and educators can guide digital screen exposure, enhance the quality of the parent–child relationship, and foster peer engagement. Preschools can incorporate digital literacy education, while policymakers may consider developing guidelines regarding digital screen exposure and content. Media producers and tech companies should aim to provide age-appropriate content and parental controls, and communities, along with public health organizations, can offer awareness programs and support services. By fostering collaboration among these stakeholders, we may help create environments supportive of children’s emotional regulation, healthy social relationships, and overall well-being in the digital age.

## Data Availability

The raw data supporting the conclusions of this article will be made available by the authors, without undue reservation.

## References

[ref1] Aishworiya R. Kiing J. S. Chan Y. H. Tung S. S. Law E. (2018). Screen time exposure and sleep among children with developmental disabilities. J. Paediatr. Child Health 54, 889–894. doi: 10.1111/jpc.13918, PMID: 29672990

[ref2] Bowen M. (1994). Family therapy in clinical practice. 1st. Northvale, N.J.: J. Aronson. Available online at: https://secure.syndetics.com/index.aspx?isbn=1568210116/summary.html&client=univnevegas&type=rn12

[ref3] Brockmann P. E. Diaz B. Damiani F. Villarroel L. Núñez F. Bruni O. (2016). Impact of television on the quality of sleep in preschool children. Sleep Med. 20, 140–144. doi: 10.1016/j.sleep.2015.06.005, PMID: 26299471

[ref4] Bustamante J. C. Fernández-Castilla B. Alcaraz-Iborra M. (2023). Relation between executive functions and screen time exposure in under 6 year-olds: a meta-analysis. Comput. Hum. Behav. 145:107739. doi: 10.1016/j.chb.2023.107739

[ref5] Carneiro A. Dias P. Pinto R. Baião R. Mesquita A. Soares I. (2019). Agreement and disagreement on emotional and behavioral problems in a sample of preschool-age children. J. Psychoeduc. Assess. 37, 154–168. doi: 10.1177/0734282917736392

[ref6] Cerniglia L. Cimino S. Ammaniti M. (2021). What are the effects of screen time on emotion regulation and academic achievements? A three-wave longitudinal study on children from 4 to 8 years of age. J. Early Child. Res. 19, 145–160. doi: 10.1177/1476718X20969846

[ref7] Cliff D. P. Howard S. J. Radesky J. S. McNeill J. Vella S. A. (2018). Early childhood media exposure and self-regulation: bidirectional longitudinal associations. Acad. Pediatr. 18, 813–819. doi: 10.1016/j.acap.2018.04.012, PMID: 29704999

[ref8] Coyne S. M. Holmgren H. G. Shawcroft J. E. Barr R. Davis E. Ashby S.. (2022). ABCs or attack–boom–crash? A longitudinal analysis of associations between media content and the development of problematic media use in early childhood. Technol. Mind Behav. 3:1037. doi: 10.1037/tmb0000093, PMID: 37908683 PMC10617637

[ref9] Coyne S. M. Reschke P. J. Stockdale L. Gale M. Shawcroft J. Gentile D. A.. (2023). Silencing screaming with screens: the longitudinal relationship between media emotion regulation processes and children’s emotional reactivity, emotional knowledge, and empathy. Emotion 23, 2194–2204. doi: 10.1037/emo0001222, PMID: 37053409 PMC10570398

[ref10] Curby T. W. Zinsser K. M. Gordon R. A. Ponce E. Syed G. Peng F. (2022). Emotion-focused teaching practices and preschool children’s social and learning behaviors. Emotion 22, 1869–1885. doi: 10.1037/emo0000988, PMID: 34726429

[ref11] Durkin K. (2010) in Handbook of peer interactions, relationships, and groups. eds. Rubin K. H. Bukowksi W. M. Laursen B., vol. 40. New York, NY: The Guilford Press.

[ref12] Eirich R. McArthur B. A. Anhorn C. McGuinness C. Christakis D. A. Madigan S. (2022). Association of Screen Time with Internalizing and Externalizing Behavior Problems in children 12 years or younger: a systematic review and Meta-analysis. JAMA Psychiatry 79, 393–405. doi: 10.1001/jamapsychiatry.2022.0155, PMID: 35293954 PMC8928099

[ref13] Evans K. E. Schmidt-Sane M. M. Bender A. E. Berg K. A. Holmes M. R. (2022). Children’s exposure to intimate partner violence and acceptance or appraisals of IPV: a systematic review. J. Fam. Violence 37, 1301–1319. doi: 10.1007/s10896-021-00318-w

[ref14] Goodman R. (1997). The strengths and difficulties questionnaire: a research note. J. *Child Psychol. Psychiatry* 38, 581–586. doi: 10.1111/j.1469-7610.1997.tb01545.x, PMID: 9255702

[ref15] Guellai B. Somogyi E. Esseily R. Chopin A. (2022). Effects of screen exposure on young children’s cognitive development: a review. Front. Psychol. 13:923370. doi: 10.3389/fpsyg.2022.923370, PMID: 36059724 PMC9431368

[ref16] Guerrero M. D. Barnes J. D. Chaput J.-P. Tremblay M. S. (2019). Screen time and problem behaviors in children: exploring the mediating role of sleep duration. Int. J. Behav. Nutr. Phys. Act. 16:105. doi: 10.1186/s12966-019-0862-x, PMID: 31727084 PMC6854622

[ref17] Hinkley T. Verbestel V. Ahrens W. Lissner L. Molnár D. Moreno L. A.. (2014). Early childhood electronic media use as a predictor of poorer well-being: a prospective cohort study. JAMA Pediatr. 168, 485–492. doi: 10.1001/jamapediatrics.2014.94, PMID: 24639016

[ref18] Huang P. Chan S. Y. Ngoh Z. M. Ong Z. Y. Low X. Z. Law E. C.. (2024). Screen time, brain network development and socio-emotional competence in childhood: moderation of associations by parent–child reading. Psychol. Med. 54, 1992–2003. doi: 10.1017/S0033291724000084, PMID: 38314509 PMC11413359

[ref19] Kabali H. K. Irigoyen M. M. Nunez-Davis R. Budacki J. G. Mohanty S. H. Leister K. P.. (2015). Exposure and use of Mobile media devices by young children. Pediatrics 136, 1044–1050. doi: 10.1542/peds.2015-2151, PMID: 26527548

[ref20] Kahn M. Schnabel O. Gradisar M. Rozen G. S. Slone M. Atzaba-Poria N.. (2021). Sleep, screen time and behaviour problems in preschool children: an actigraphy study. Eur. Child Adolesc. Psychiatry 30, 1793–1802. doi: 10.1007/s00787-020-01654-w, PMID: 33006004

[ref21] Kaur N. Gupta M. Kiran T. Malhi P. Grover S. (2021). Development and evaluation of the digital-screen exposure questionnaire (DSEQ) for young children. PLoS One 16:e0253313. doi: 10.1371/journal.pone.0253313, PMID: 34157053 PMC8219135

[ref22] Keşşafoğlu D. Küntay A. Uzundağ B. A. (2024). Immediate and delayed effects of fantastical content on children’s executive functions and mental transformation. J. Exp. Child Psychol. 248:106067. doi: 10.1016/j.jecp.2024.106067, PMID: 39241323

[ref23] Klein A. M. Otto Y. Fuchs S. Reibiger I. Von Klitzing K. (2015). A prospective study of behavioral and emotional symptoms in preschoolers. Eur. Child Adolesc. Psychiatry 24, 291–299. doi: 10.1007/s00787-014-0575-2, PMID: 24972693

[ref24] Laugen N. J. Kårstad S. B. Reinfjell T. Wichstrøm L. (2024). The development of emotion understanding in children: the importance of parents, teachers, and peers. Dev. Psychol. 60, 255–264. doi: 10.1037/dev0001627, PMID: 37733000

[ref25] Lindsey E. W. (2017). Mutual positive emotion with peers, emotion knowledge, and Preschoolers’Peer acceptance. Soc. Dev. 26, 349–366. doi: 10.1111/sode.12201

[ref26] Liu X. Geng S. Dou D. (2024). Interplay between Children's electronic media use and prosocial behavior: the chain mediating role of parent-child closeness and emotion regulation. Behav. Sci. 14:436. doi: 10.3390/bs14060436, PMID: 38920768 PMC11200768

[ref27] Liu W. Wu X. Huang K. Yan S. Ma L. Cao H.. (2021). Early childhood screen time as a predictor of emotional and behavioral problems in children at 4 years: a birth cohort study in China. Environ. Health Prev. Med. 26:3. doi: 10.1186/s12199-020-00926-w, PMID: 33413099 PMC7789634

[ref28] Livingstone S. Mascheroni G. Staksrud E. (2018). European research on children’s internet use: assessing the past and anticipating the future. New Media Soc. 20, 1103–1122. doi: 10.1177/1461444816685930

[ref29] Lu W. Li X. (2022). Are screens raising ‘problem children’? --a meta-analysis of the relationship between screen exposure and preschool children’s problem behaviours. Res. Pre School Educ. 25, 49–61. doi: 10.13861/j.cnki.sece.2022.06.009

[ref30] Ma X. Zhang Y. (2020). Language, screen exposure and peer relation problems in preschool children. J. Dev. Behav. Pediatr. 41, 25–28. Available online at: https://webofscience-clarivate-cn-s.vpn.muc.edu.cn:8118/wos/alldb/full-record/WOS:000526852800020

[ref31] Montagni I. Guichard E. Kurth T. (2016). Association of screen time with self-perceived attention problems and hyperactivity levels in French students: a cross-sectional study. BMJ Open 6:e009089. doi: 10.1136/bmjopen-2015-009089, PMID: 26920440 PMC4769424

[ref32] Morrison F. J. Grammer J. K. (2016). “Conceptual clutter and measurement mayhem: proposals for cross-disciplinary integration in conceptualizing and measuring executive function” in Executive function in preschool-age children: Integrating measurement, neurodevelopment, and translational research. eds. Griffin J. A. McCardle P. Freund L. S. (Washington: American Psychological Association), 327–348.

[ref33] Ortega-Mohedano F. Pinto-Hernández F. (2021). Predicting wellbeing in children’s use of smart screen devices. Comunicar 29, 119–128. doi: 10.3916/C66-2021-10

[ref34] Pianta R. C. (1994). Patterns of relationships between children and kindergarten teachers. J. Sch. Psychol. 32, 15–31. doi: 10.1016/0022-4405(94)90026-4

[ref35] Przybylski A. K. Weinstein N. (2019). Digital screen time limits and young Children’s psychological well-being: evidence from a population-based study. Child Dev. 90, e56–e65. doi: 10.1111/cdev.13007, PMID: 29235663

[ref39] Shoshani A. Nelke S. Girtler I. (2022). Tablet applications as socializing platforms: the effects of prosocial touch screen applications on young children’s prosocial behavior. Comput. Hum. Behav. 127:107077. doi: 10.1016/j.chb.2021.107077

[ref40] Skalická V. Wold Hygen B. Stenseng F. Kårstad S. B. Wichstrøm L. (2019). Screen time and the development of emotion understanding from age 4 to age 8: a community study. Br. J. Dev. Psychol. 37, 427–443. doi: 10.1111/bjdp.12283, PMID: 30816568

[ref41] Slater M. D. (2004). Operationalizing and analyzing exposure: the Foundation of Media Effects Research. Journal. Mass Commun. Q. 81, 168–183. doi: 10.1177/107769900408100112

[ref42] Wu X. Tao S. Rutayisire E. (2017). The relationship between screen time, nighttime sleep duration, and behavioural problems in preschool children in China. Eur. Child Adolesc. Psychiatry 26, 541–548. doi: 10.1007/s00787-016-0912-8, PMID: 27822641

[ref43] Wu Z. Yu J. Xu C. (2023). Does screen exposure necessarily relate to behavior problems? The buffering roles of emotion regulation and caregiver companionship. Early Child. Res. Q. 63, 424–433. doi: 10.1016/j.ecresq.2023.01.008

[ref44] You J. Lv M. Yingyu Y. Chen Y. (2023). Effects of screen exposure on early childhood development. Chin. J. Mater. Child Health 14, 65–69. doi: 10.19757/j.cnki.issn1674-7763.2023.02.013

